# Molecular basis of fruit development

**DOI:** 10.3389/fpls.2015.00028

**Published:** 2015-02-05

**Authors:** Zhongchi Liu, Robert G. Franks

**Affiliations:** ^1^Department of Cell Biology and Molecular Genetics, University of MarylandCollege Park, MD, USA; ^2^Department of Plant and Microbial Biology, North Carolina State UniversityRaleigh, NC, USA

**Keywords:** fruit evolution, endocarp, auxin, cytokinin, morphogenesis, ovule, gynoecium, carpel margin meristem

The fruit is a vital plant structure that supports seed development and dispersal, and is an indispensable part of the human diet. The 11 articles within this special research topic focus on the molecular mechanisms of early fruit development and span a diversity of species and experimental approaches. Since the gynoecium, the female floral structure, is the precursor of all or part of the fruit, several articles are focused on mechanisms of gynoecium development. The articles can be organized into several groups based on common themes highlighted below.

## Patterning of the gynoecium

The gynoecium consists of one to several carpels, usually fused together and topped with style and stigma. The botanical fruit is derived from the carpel wall (pericarp) and genes that regulate gynoecium development ultimately affect fruit size, shape, and dispersal mode. Hence, one could not discuss fruit development without understanding the mechanism controlling the gynoecium development.

The gynoecium is a three dimensional structure with three positional axes: basal-apical; medial-lateral; and abaxial-adaxial. Auxin synthesis, transport, and signaling have been implicated in the regulation of all three axes. Previously, the auxin gradient model (Nemhauser et al., 2000) proposed that auxin was synthesized at the apical tip of the gynoecium and then transported basally, forming a gradient from high auxin concentrations at the apex to low concentrations at the base. The differential cellular responses to the auxin gradient resulted in the apical-basal patterning of the gynoecium.

In this research topic, Zuniga-Mayo et al. (2014) added a new dimension to this model by showing that exogenous cytokinin application (benzyl amino purine, BAP) to the *Arabidopsis* inflorescence caused a phenotype similar to that caused by the auxin transport inhibitor NPA (1-*N*-naphtylphtalamic acid). Hence, cytokinin may reduce auxin transport and thus also be involved in gynoecium apical-basal patterning.

Hawkins and Liu (2014) proposed an alternative model in lieu of the auxin gradient model. They pointed out that the gynoecium apical-basal axis determination likely occurred very early, long before auxin biosynthesis occurs at the apical tip. This new model suggests that, much like leaf patterning, it is the role of auxin in the abaxial-adaxial polarity establishment that determines proper apical to basal patterning of the gynoecium.

At later stages of the gynoecium development, auxin biosynthesis at the apex may be critical to formation of the style and stigma. In *NGATHA* (*NGA*) gain- or loss-of-function mutants of *Arabidopsis*, when apical development is disrupted, Martinez-Fernandez et al. (2014) identified 2449 genes whose expression levels were altered. Their analysis of these genes suggests that the NGA proteins regulate gynoecial development via the control of auxin homeostasis.

## Carpel margin meristems (CMMs) and shoot apical meristem (SAM) are regulated with similar mechanisms

Carpel margin meristems (CMMs) are the meristematic medial portions of the gynoecium that give rise to the ovules. Arnaud and Pautot (2014) reviewed the roles of TALE HD (three amino acid loop extension homeodomain) transcription factors in regulating CMMs of *Arabidopsis*, highlighting similar molecular mechanisms underlying CMM and SAM (shoot apical meristem) with respect to gibberellic acid and cytokinin signaling. Another similarity between the CMM and the SAM was highlighted by Kamiuchi et al. (2014) as they defined the role of *CUP-SHAPED COTYLEDON1* (*CUC1*) and *CUC2* genes in initiating and positioning the CMM and in the regulation of *SHOOTMERISTEMLESS* (*STM*) expression in the gynoecium. Wynn et al. (2014) reveal a role for the transcription factor *PERIANTHIA* (*PAN*) during CMM development as well as during floral meristem determinancy. Their work suggested that proper termination of the floral meristem may be required for the complete development of the CMM and ovules.

Cucinotta et al. (2014) reviewed the early ovule development with a focus on the formation of the CMM and the initiation of ovule primordia in *Arabidopsis*. They presented a model of ovule initiation that relates the functions of *CUC* and *ANT* genes as well as the action of auxin, cytokinin, and brassinosteroid hormones (Galbiati et al., 2013; Cucinotta et al., 2014). Their model posits a role for *ANT* in the growth of the organ primordia, and *CUC* genes in the specification of the boundary zones between ovules. The interactions between the primordial and boundary regions, as well as cytokinin regulation are required for the proper expression and localization of the *PIN-FORMED1* (*PIN1*) auxin transporter and thus for proper auxin fluxes. Brassinosteroid signaling is proposed to support ovule initiation through the stimulation of *ANT* activity in ovule primordia (Huang et al., 2013; Cucinotta et al., 2014).

## Fruit shape, size, and ripening

Two articles each provided a unique perspective on fruit development and ripening. Van Der Knaap et al. (2014) discussed six key genes and their mechanisms that regulate tomato fruit shape and weight. Some of the genes also act during floral meristem and floral organ development, highlighting the close connection between floral organ initiation, specification and later fruit shape and sizes.

The review by Pesaresi et al. (2014) focused on the retrograde (plastids to nucleus) and anterograde (nucleus to plastids) communication pathways during fruit ripening, areas that were not yet fully explored. In the anterograde pathway, nuclear-encoded regulators alter plastid function and specify plastid types (Leon et al., 1998; Raynaud et al., 2007). The retrograde pathways allow for the transfer of information from the plastid to the nucleus regarding the functional or physiological status of the plastids (Chi et al., 2013).

## Evolutionary perspectives of fruit development

Upon fertilization, the carpel in many species transitions from an ovule-containing vessel to the seed containing fruit. Dardick and Callahan (2014) reviewed molecular mechanisms that regulate endocarp differentiation in each of three species from the families Brassicaceae, Rosaceae, and Solanaceae. The endocarp is the innermost cell layer of the carpel wall. They discussed in detail current understanding of the “stone” endocarp in peach and suggested that the regulatory genes and pathways controlling the lignified valve margin layer of Brassica's dry fruit are similar to those controlling lignified “stone” endocarp in peach.

Pabon-Mora et al. (2014) took a phylogenetic approach to analyze the transcription factor genes that regulate carpel valve margins of dry fruit in Arabidopsis. Through comprehensive searches for homologs across core-eudicots, basal eudicots, monocots, and basal angiosperms and phylogenetic tree construction, the authors suggested conservation of certain fruit development pathways and established the foundation for future functional tests.

## Summary

The diverse perspectives presented in this research topic provide an in depth understanding of ongoing researches in this exciting and evolving field. One common theme emerging from several articles is that distinct structures do not always result from entirely distinct regulatory networks, (e.g., similar genes regulate SAM, FM, and CMM development, similar mechanisms may underlie patterning of carpels and leaves, and conserved networks are required for the stone endocarp in peach and the dry fruit valve margin in Brassica). Because of the broad biological questions addressed as well as the potential applications to agricultural problems, this field will likely attract further interest and funding, and yield important discoveries in the future.

### Conflict of interest statement

The authors declare that the research was conducted in the absence of any commercial or financial relationships that could be construed as a potential conflict of interest.
